# Interpreting Near‐Threshold Age‐Adjusted D‐Dimer for Suspected Pulmonary Embolism in the Emergency Department: A Retrospective Diagnostic Accuracy Study

**DOI:** 10.1155/emmi/8353356

**Published:** 2026-07-16

**Authors:** Cem Yıldırım, Ahmet Fırat Bektaş, Ertuğ Günsoy, Ahmet Aykut, Vedat Kırpat, Mehmet Veysel Öncül

**Affiliations:** ^1^ Emergency Department, Van Education and Research Hospital, Van, Türkiye, vai.org; ^2^ Emergency Department, Akdeniz University Hospital, Antalya, Türkiye

**Keywords:** age-adjusted D-dimer, D-dimer, diagnostic accuracy, emergency department, near-threshold, pulmonary embolism

## Abstract

**Background:**

Age‐adjusted D‐dimer (AAD) thresholds are used to reduce false positives in older patients evaluated for pulmonary embolism (PE). However, evidence is limited regarding test behavior near the individualized cutoff (“near‐threshold” zone) and the safety of excluding PE in AAD‐negative cases.

**Methods:**

We conducted a retrospective diagnostic accuracy study of adults ≥ 50 years presenting to a tertiary emergency department (2015–2019) with suspected PE who underwent D‐dimer testing and definitive imaging (CT pulmonary angiography or V/Q scintigraphy). Diagnostic performance of the standard threshold (0.5 µg/mL, FEU) and AAD (age ×  0.01 µg/mL) was assessed using ROC analysis. Accuracy metrics are reported with 95% confidence intervals (CIs) via bootstrap; AUC CIs and between‐curve comparisons used the DeLong method. The near‐threshold zone was defined as ±0.1 µg/mL around the AAD cutoff. Clinical charts of AAD‐negative PE cases were reviewed.

**Results:**

Among 979 patients, 162 (16.5%) had imaging‐confirmed PE. Standard D‐dimer showed sensitivity 99.4% (95% CI 98.0–100.0) and specificity 4.0% (2.7–5.4); AAD showed sensitivity 97.5% (94.8–99.4) and specificity 11.8% (9.6–13.9). AUCs were 0.734 and 0.727, with no significant difference (DeLong *p* = 0.41). Within the near‐threshold window, PE prevalence was 7.7%, increasing with age (28.6% in ≥ 80 years). Three patients had PE despite AAD‐negative results; all had segmental or subsegmental emboli, and no in‐hospital complications were documented in the available records.

**Conclusion:**

In this imaging‐selected retrospective cohort, AAD improved specificity with minimal sensitivity loss. However, AAD should not be used as a stand‐alone rule‐out strategy and should be applied only within clinical probability‐guided diagnostic pathways. Near‐threshold results are not risk‐free, particularly in older adults, and should prompt careful clinical reassessment when overall probability remains concerning.

## 1. Introduction

Pulmonary embolism (PE) is a potentially life‐threatening condition and remains a frequent diagnostic challenge in emergency departments (EDs). The 2019 European Society of Cardiology (ESC) guidelines emphasize the importance of a structured diagnostic approach combining clinical assessment, D‐dimer testing, and imaging to ensure both diagnostic accuracy and resource efficiency [1]. Given its broad and often nonspecific presentation, PE may mimic various cardiopulmonary conditions, leading to a high rate of imaging studies, many of which ultimately yield negative results [2].

To reduce unnecessary imaging in low‐risk patients, clinical decision rules such as the Wells score and the Pulmonary Embolism Rule‐out Criteria (PERC) have been developed and widely implemented [3, 4]. Another validated approach is the YEARS algorithm, which combines D‐dimer testing with three clinical variables: hemoptysis, signs of deep vein thrombosis (DVT), and whether PE is the most likely diagnosis. In patients with no positive YEARS criteria, a higher D‐dimer threshold of 1.0 µg/mL may be applied, allowing more patients to avoid imaging [5]. Although YEARS has demonstrated safety and efficiency in large prospective studies, its reliance on structured clinical scoring may limit feasibility in retrospective analyses or routine practice settings.

For patients with low or intermediate clinical probability, D‐dimer testing is often used as a noninvasive method to exclude PE. While the test offers high sensitivity, its specificity declines significantly with increasing age, resulting in a substantial number of false positives in older populations [6].

To improve specificity without compromising safety, an age‐adjusted D‐dimer (AAD) threshold has been proposed, typically calculated as age × 0.01 µg/mL in patients aged 50 years and above. Initial validation studies, including a retrospective analysis of three large cohorts, showed that AAD increases the proportion of patients in whom PE can be safely excluded without imaging [6]. This finding was later confirmed by a large meta‐analysis, further supporting the clinical utility of age adjustment in elderly populations [7].

Despite growing acceptance of AAD in emergency care, uncertainties remain regarding its performance near the individualized threshold—particularly in patients whose D‐dimer results fall just below the cutoff. This “borderline zone” has not been clearly defined or systematically studied and may represent an area of diagnostic vulnerability in real‐world practice.

This study aimed to evaluate the diagnostic performance of age‐adjusted versus standard D‐dimer thresholds in patients aged ≥ 50 years presenting with suspected PE, with specific attention to the borderline zone and AAD‐negative PE cases.

## 2. Methods

### 2.1. Study Design and Setting

This retrospective diagnostic accuracy study was conducted at the ED of a tertiary academic medical center in Türkiye, with approximately 150,000 annual ED visits. The study period covered patients who presented between January 1, 2015, and December 31, 2019, with suspected PE and underwent D‐dimer testing. The primary aim was to compare the diagnostic performance of standard and AAD thresholds, with particular attention to a “borderline zone” near the individualized cutoff. The study was reported in accordance with the Standards for Reporting Diagnostic Accuracy Studies (STARD) framework.

### 2.2. Inclusion and Exclusion Criteria

Patients were eligible for inclusion if they met all of the following criteria.

• Age ≥ 50 years
• D‐dimer testing performed for suspected PE
• Advanced imaging with either computed tomography pulmonary angiography (CTPA) or ventilation/perfusion (V/Q) scintigraphy

Patients were excluded if they had.

• No imaging performed (either due to clinical decision or early discharge)
• Multiple ED visits during the study period (only the first visit was included)
• Incomplete data (e.g., missing D‐dimer result or imaging report)

Of the 2183 patients initially evaluated, 1204 were excluded—primarily due to the absence of definitive imaging, which precluded diagnostic accuracy assessment as required for the study’s primary aim—resulting in a final cohort of 979 patients with both D‐dimer results and completed imaging studies. Because definitive imaging was a prerequisite for inclusion, the final cohort likely represents patients with higher clinical suspicion for PE than an unselected ED population. This imaging‐only design may introduce verification and spectrum bias, which can affect estimates of sensitivity, specificity, and predictive values.

### 2.3. Data Collection and Variables

Demographic variables (age and sex), clinical presentation, D‐dimer levels, and imaging modality and findings were retrospectively extracted from the hospital’s electronic health records by trained data abstractors using a standardized data collection protocol. Abstractors were not blinded to the study hypothesis, and no duplicate independent data extraction was performed. PE was defined according to the original clinical radiology reports indicating intravascular filling defects on CTPA or matched perfusion defects on V/Q scans. Imaging studies were not centrally re‐reviewed for the purposes of this study. Therefore, although the primary outcome was based on objective imaging reports generated during routine clinical care, some degree of misclassification bias may have occurred, particularly for small, subsegmental, indeterminate, or borderline imaging findings.

Information on therapeutic anticoagulation before ED blood draw (e.g., DOACs, warfarin, or heparin within 24 h) was not consistently documented across medication reconciliation and administration records and therefore was neither used as an exclusion criterion nor adjusted for in the analyses.

### 2.4. D‐Dimer Testing and Adjustment

Quantitative D‐dimer assays were performed as part of standard care using immunoturbidimetric methods, and results were reported in μg/mL fibrinogen equivalent units (FEU).

The standard D‐dimer cutoff was defined as 0.5 µg/mL.

The AAD threshold was calculated as age × 0.01 µg/mL for patients ≥ 50 years of age, in line with prior validation studies [5].

Diagnostic accuracy metrics (sensitivity, specificity, predictive values, and likelihood ratios) were calculated using conventional threshold‐based classification with the standard D‐dimer cutoff (0.5 µg/ml, FEU) and the age‐adjusted threshold (age × 0.01 µg/ml for patients ≥ 50 years). For receiver operating characteristic (ROC) analysis, raw D‐dimer values were used to evaluate overall discrimination. In addition, to enable a graphical ROC comparison of the age‐adjusted approach on a common scale, we constructed an age‐normalized value by expressing each D‐dimer result relative to the patient’s age‐adjusted threshold (i.e., the difference between the measured D‐dimer and the individual AAD cutoff). This ROC analysis was performed for comparative visualization and did not alter the threshold‐based diagnostic accuracy calculations.

### 2.5. Definition of the Borderline Zone

To assess diagnostic uncertainty near the individualized threshold, we defined a “borderline zone” as any D‐dimer result within ±0.1 µg/mL of the patient’s age‐adjusted cutoff. This window was selected a priori as a pragmatic range intended to capture results close to the individualized decision boundary, where clinical interpretation is most uncertain, and to approximate analytical or reporting variation near the cutoff. Subgroup analysis was conducted in this population to determine PE prevalence and characteristics. Additional sensitivity analyses using alternative borderline‐zone thresholds were not performed because this analysis was exploratory and included a limited number of PE events, making narrower or wider threshold‐based subgroups potentially unstable.

### 2.6. AAD‐Negative PE Subgroup

All patients who had imaging‐confirmed PE despite having D‐dimer values below their AAD threshold were identified. For this subset, clinical records were reviewed to determine the PE location (e.g., segmental or subsegmental), initial treatment, and short‐term clinical outcome during the index ED visit.

### 2.7. Statistical Analysis

Continuous variables were summarized using mean ± standard deviation (SD) or median and interquartile range (IQR), depending on normality. Categorical variables were reported as counts and percentages. Comparisons between PE and non‐PE groups were made using the Student’s *t*‐test or Mann–Whitney *U* test for continuous variables and the chi‐square test or Fisher’s exact test for categorical variables, as appropriate.

Diagnostic performance for the standard and age‐adjusted thresholds was assessed using ROC analysis. We calculated AUC, sensitivity, specificity, PPV, NPV, and LR+ and LR−. Diagnostic accuracy measures (sensitivity, specificity, PPV, NPV, LR+, and LR−) are reported with 95% confidence intervals (CIs) derived by nonparametric bootstrap (5000 resamples). ROC curves were compared using the DeLong method. A *p* value < 0.05 was considered statistically significant. No formal sample size or power calculation was performed because this retrospective study included all eligible patients who underwent D‐dimer testing and definitive imaging during the predefined study period. Therefore, diagnostic accuracy estimates, particularly those derived from subgroup and borderline‐zone analyses, should be interpreted with attention to the width of their CIs and the exploratory nature of these analyses.

All statistical analyses were performed using Python Version 3.11 with the scikit‐learn and statsmodels libraries. Supporting analyses were cross‐validated using SPSS Version 29.0 (IBM Corp, Armonk, NY).

### 2.8. Ethical Considerations

This study was approved by the Clinical Research Ethics Committee of Akdeniz University (Approval No: KAEK‐260, dated April 8, 2020). As a retrospective study using anonymized patient data, the requirement for informed consent was waived. The study adhered to the principles outlined in the Declaration of Helsinki. Trial registration was not required because this was a retrospective observational diagnostic accuracy study using anonymized existing data.

## 3. Results

### 3.1. Study Population

**FIGURE 1:**
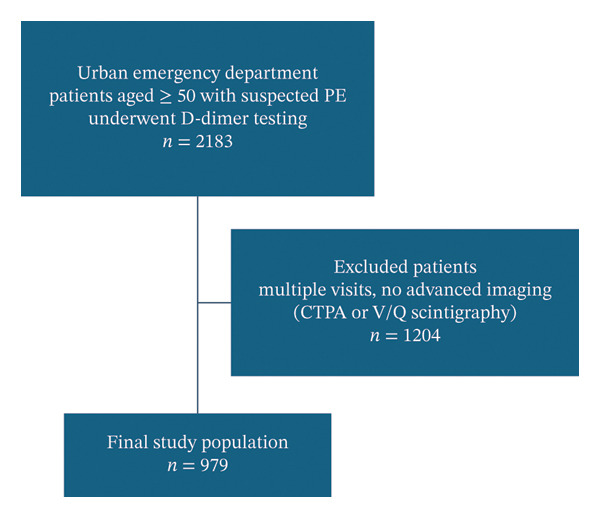
Patient flowchart. This flowchart shows the selection process of the study population. A total of 2183 patients aged ≥ 50 years who underwent D‐dimer testing for suspected PE were screened. 1204 patients were excluded due to multiple visits or lack of advanced imaging. The final study population included 979 patients who underwent both D‐dimer testing and imaging.

Initially, 2183 patients aged 50 years and older who underwent D‐dimer testing for suspected PE were screened. Of these, 1204 were excluded due to repeated ED visits or lack of advanced imaging (CTPA or V/Q scintigraphy), as shown in Figure 1. The final study population included 979 patients who met the inclusion criteria and underwent both D‐dimer testing and advanced imaging. The baseline characteristics, imaging findings, and diagnostic accuracy measures were analyzed to compare standard and AAD thresholds in this imaging‐selected cohort.

### 3.2. Patient Characteristics

A total of 979 patients were included in the study, of whom 162 (16.5%) were diagnosed with PE, while 817 (83.5%) were in the non‐PE group. The mean age of patients with PE was 67.6 ± 10.8 years, which was significantly higher than the non‐PE group (64.9 ± 10.1 years, *p* = 0.002). Among all participants, 61.4% (502/817) of the non‐PE group and 51.9% (84/162) of the PE group were female, showing a statistically significant difference in gender distribution between groups (*p* = 0.023) (Table 1).

**TABLE 1:** Demographic and clinical characteristics of patients with and without pulmonary embolism (PE).

*n* = 979	Non‐PE (817)		PE (162)		*p*
	X̄	±	X̄	±	
Age	64.9	10.1	67.6	10.8	0.002

	***n***	**%**	***n***	**%**	

Gender (f/m)	502/315	61.4	84/78	51.9	0.023
CTPA (875)	767	87.7	108	12.3	
V/Q (138)	79	57.2	59	42.8	

*Note:* PE: pulmonary embolism group. Non‐PE: patients without pulmonary embolism. V/Q scintigraphy: ventilation/perfusion scintigraphy. *p* value: Statistical significance level; values < 0.05 indicate a significant difference. Mean ± SD: Data are presented as mean ± standard deviation. The chi‐square test was used for categorical variables and the Student’s *t*‐test for normally distributed continuous variables.

Abbreviation: CTPA, computed tomography pulmonary angiography.

### 3.3. Imaging Modalities and PE Diagnosis

Of the 979 patients, 875 (89.4%) underwent CTPA, and 138 (14.1%) underwent V/Q scintigraphy. Among those who underwent CTPA, 12.3% (108/875) had a confirmed PE diagnosis, while among patients who underwent V/Q scintigraphy, 42.8% (59/138) had a positive result for PE (Table 1). A small subset of patients (*n* = 35) underwent both CTPA and V/Q scintigraphy, usually when the initial study was nondiagnostic or technically limited. For the purposes of this analysis, the final imaging modality was considered the reference standard. In addition, V/Q scintigraphy was used in patients with contraindications to iodinated contrast (e.g., allergy or renal impairment), which—together with its use after nondiagnostic CTPA—explains its relatively high frequency in this cohort.

### 3.4. D‐Dimer and AAD Levels in PE vs. Non‐PE Groups

As presented in Table 2, median levels of both standard D‐dimer and AAD‐transformed D‐dimer were significantly higher in patients diagnosed with PE compared with those without PE. The median D‐dimer level in the PE group was 3.00 µg/mL (IQR, 1.35–5.62), whereas it was 1.14 µg/mL (0.75–2.02) in the non‐PE group (*p* < 0.001).

**TABLE 2:** Comparison of D‐dimer and age‐adjusted D‐dimer levels between PE and non‐PE groups.

*n* = 979	Non‐PE (817)			PE (162)			*p*
	Median	Q1	Q3	Median	Q1	Q3	
D‐dimer (μg/mL, FEU)	1.14	0.75	2.02	3.00	1.35	5.62	< 0.001
AAD (μg/mL, FEU)	0.99	0.640	1.18	2.73	1.18	5.46	< 0.001

*Note:* PE: pulmonary embolism group. Non‐PE: patients without pulmonary embolism. D‐dimer: plasma D‐dimer level; AAD: age‐adjusted D‐dimer. Median (Q1–Q3): Data are presented as median and interquartile range. The Mann–Whitney *U* test was used for comparisons.

### 3.5. Overlap Between D‐Dimer Negativity, AAD Negativity, and PE Diagnosis

**FIGURE 2:**
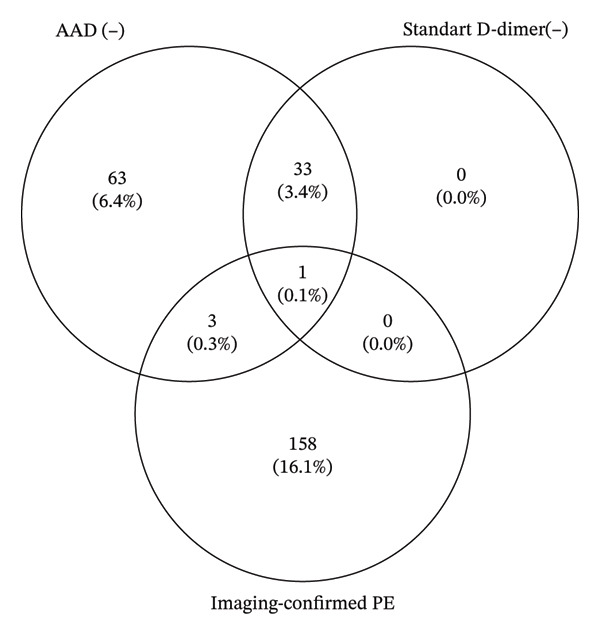
Overlap between age‐adjusted D‐dimer negativity, standard D‐dimer negativity, and imaging‐confirmed pulmonary embolism. The Venn diagram illustrates the overlap between patients with negative age‐adjusted D‐dimer results, negative standard D‐dimer results (< 0.5 µg/mL), and imaging‐confirmed pulmonary embolism.

As shown in Figure 2, there is an overlap between standard D‐dimer negativity, AAD negativity, and imaging‐confirmed PE diagnoses. Among the 162 patients diagnosed with PE, 161 (99.4%) had D‐dimer values ≥ 0.5 µg/mL before adjustment, while 158 (97.5%) remained above the age‐adjusted threshold. Three PE patients who initially tested positive by the standard threshold became negative after age adjustment, and one patient tested negative with both methods.

### 3.6. ROC Curve and Diagnostic Performance

**FIGURE 3:**
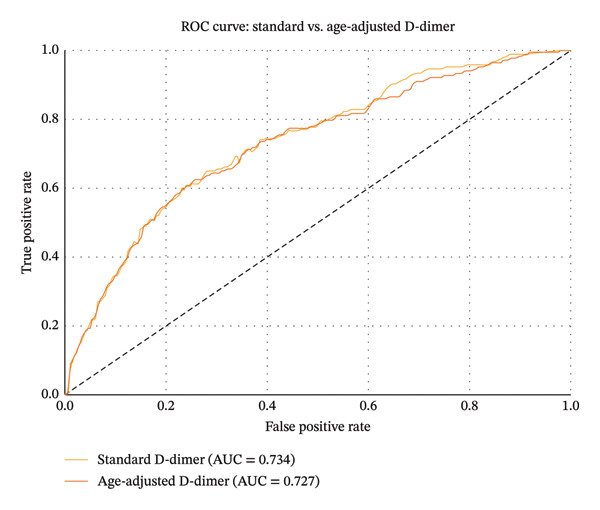
Receiver operating characteristic (ROC) curves for D‐dimer and age‐adjusted D‐dimer in predicting pulmonary embolism. Receiver operating characteristic (ROC) curves compare overall discrimination of the standard and age‐adjusted approaches for imaging‐confirmed PE. AUCs were 0.734 and 0.727, with no significant difference between curves (DeLong *p* = 0.41).

The ROC curves demonstrated moderate overall discrimination between the standard and age‐adjusted approaches, with areas under the curve (AUC) of 0.734 and 0.727, respectively (Figure 3 and Table 3). The between‐curve difference was not significant (DeLong *p* = 0.41).

**TABLE 3:** Diagnostic performance of D‐dimer and age‐adjusted D‐dimer in predicting pulmonary embolism.

Cutoff	Non‐PE (817)		PE (162)		AUC (95% CI)	*p*
	*n*	%	*n*	%		
D‐dimer						
< 0.5 µg/mL (FEU)	33	4.0	1	0.6	0.734 (0.609–0.859)	< 0.001
≥ 0.5 µg/mL (FEU)	784	96.0	161	99.4		
AAD						
< Age‐adjusted threshold	96	11.8	4	2.5	0.727 (0.599–0.850)	< 0.001
≥ Age‐adjusted threshold	721	88.2	158	97.5		

*Note:* PE: pulmonary embolism group, Non‐PE: patients without pulmonary embolism, D‐dimer: plasma D‐dimer level. AAD: age‐adjusted D‐dimer. Cutoff values indicate the threshold levels for classification. Data are presented as absolute numbers and percentages. For AAD, the cutoff is age‐dependent (age × 0.01 µg/mL, FEU, for patients ≥ 50 years).

Abbreviation: AUC, area under the curve.

### 3.7. Sensitivity, Specificity, and Likelihood Ratios

Diagnostic accuracy estimates with 95% CIs are shown in Table 4. Briefly, standard D‐dimer yielded a sensitivity of 99.4% (95% CI, 98.0–100.0) and specificity of 4.0% (2.7–5.4), whereas AAD showed a sensitivity of 97.5% (94.8–99.4) and specificity of 11.8% (9.6–13.9). The negative likelihood ratio was 0.15 (< 0.01–0.57) for standard D‐dimer versus 0.21 (0.05–0.45) for AAD, while the positive likelihood ratio improved slightly with AAD—1.11 (1.07–1.14) versus 1.04 (1.02–1.05). PPV and NPV are provided in Table 4.

**TABLE 4:** Diagnostic accuracy measures of D‐dimer and age‐adjusted D‐dimer for pulmonary embolism.

*n* = 979	Cutoff	%				NLR	PLR
		Sens.	Spec.	PPV	NPV		
D‐dimer	≥ 0.5 µg/mL	99.4 (98.0–100.0%)	4.0 (2.7–5.4%)	17.0 (14.6–19.5%)	97.1 (90.0–100.0%)	0.15 (< 0.01–0.57)	1.04 (1.02–1.05)
AAD	Age × 0.01 µg/mL	97.5 (94.8–99.4%)	11.8 (9.6–13.9%)	18.0 (15.5–20.5%)	96.0 (91.8–99.1%)	0.21 (0.05–0.45)	1.11 (1.07–1.14)

*Note:* Values are point estimates with 95% confidence intervals (CIs) in parentheses. PE: pulmonary embolism group. AAD: age‐adjusted D‐dimer, Non‐PE: patients without pulmonary embolism. D‐dimer: plasma D‐dimer level. Sens.: sensitivity. Spec.: specificity. PPV: positive predictive value. NPV: negative predictive value.

Abbreviations: NLR, negative likelihood ratio, PLR, positive likelihood ratio.

### 3.8. False‐Negative Rate of AAD

Among the 979 patients included in the study, 100 individuals had D‐dimer values ≥ 0.5 µg/mL but below their age‐adjusted threshold. Within this subgroup, PE was confirmed in 3 patients (3.0%), indicating a potential false‐negative result when using AAD.

Notably, all three patients had either segmental or subsegmental PE. The first patient had a subsegmental embolism in the left lower lobe with pulmonary infarction and underlying emphysematous changes, managed as an outpatient. The second showed subsegmental thrombus and bronchitic changes, including mucus plugging, and was similarly treated without hospitalization. The third had a combined segmental‐subsegmental PE with features of bronchiolitis, for whom short‐term hospitalization was recommended. All patients were discharged on anticoagulation, and no in‐hospital complications were documented in the available records.

These findings suggest that while AAD improves specificity and reduces unnecessary imaging, it may miss small or peripheral emboli—particularly in patients with underlying pulmonary disease. In our AAD‐negative PE cases, emboli were limited to segmental or subsegmental branches, and no short‐term adverse outcomes were documented in the available records; however, longer‐term clinical safety could not be assessed.

### 3.9. Borderline Zone Analysis

To evaluate diagnostic uncertainty near the age‐adjusted threshold, a “borderline zone” was defined as D‐dimer values within ±0.1 µg/mL of the individualized AAD cutoff. A total of 130 patients (13.3%) fell into this zone. Among them, 10 (7.7%) were diagnosed with PE, significantly lower than the 17.9% positivity rate (152/849) observed in patients outside this zone (*p* = 0.004), as detailed in Table 5.

**TABLE 5:** Summary of patients in the borderline zone.

Parameter	Value
Total patients in borderline zone (n)	130
PE‐positive in borderline zone (n)	10
PE rate (%)	7.7
Median age	62
Female (%)	57.7
Median D‐dimer (μg/mL)	0.63
Median AAD threshold (μg/mL)	0.62

*Note:* Summary of demographic characteristics and PE outcomes among patients with D‐dimer values within ± 0.1 µg/mL of their age‐adjusted threshold. This group was defined as the “borderline zone” and evaluated separately due to the diagnostic ambiguity surrounding these values.

When stratified by age, the PE positivity within the borderline zone showed a notable increase: only 3.9% of patients aged 50–59 had PE, compared to 28.6% of those aged ≥ 80. This age‐related gradient in risk is illustrated in Table 6, emphasizing the need for heightened clinical vigilance when interpreting borderline D‐dimer results in elderly patients.

**TABLE 6:** PE positivity in the borderline zone by age group.

Age group	Total	PE_positive	PE_rate (%)
50–59	51	2	3.9
60–69	52	4	7.7
70–79	20	2	10.0
80 +	7	2	28.6

*Note:* Distribution of PE‐positive cases among borderline zone patients stratified by age group. Higher PE prevalence was observed in patients aged ≥ 80, suggesting that borderline values may carry increased risk in older populations.

### 3.10. Age‐Stratified AAD Performance

To further assess the diagnostic utility of AAD, we evaluated its performance across defined age brackets: 50–59, 60–69, 70–79, and ≥ 80 years. As shown in Table 7, sensitivity remained consistently above 95% in all groups, with the highest sensitivity observed in patients aged 60–69 (98.3%). Specificity remained low across all age categories, though a modest improvement was seen in patients aged 70–79, reaching 14.1%.

**TABLE 7:** Age‐stratified diagnostic performance of age‐adjusted D‐dimer (AAD) by decade groups with 95% confidence intervals.

Age group	*n*	PE +	Sensitivity	Specificity	PPV	NPV	LR+	LR−
50–59	337	46	97.8% (92.3–100.0%)	10.7% (7.2–14.4%)	14.8% (11.0–18.9%)	96.9% (89.2–100.0%)	1.09 (1.02–1.16)	0.20 (0.01–0.76)
60–69	326	47	95.7% (89.1–100.0%)	10.4% (6.9–14.2%)	15.3% (11.0–19.5%)	93.5% (83.9–100.0%)	1.07 (0.99–1.14)	0.41 (0.01–1.13)
70–79	204	42	97.6% (92.2–100.0%)	17.3% (11.7–23.2%)	23.4% (17.1–29.9%)	96.6% (88.5–100.0%)	1.18 (1.08–1.29)	0.14 (0.01–0.50)
≥ 80	112	27	100.0% (100.0–100.0%)	9.4% (3.6–16.3%)	26.0% (17.8–35.0%)	100.0% (100.0–100.0%)	1.10 (1.04–1.19)	Not estimable

*Note:* Values are point estimates with 95% CIs in parentheses; CIs were derived using nonparametric bootstrap (3000 resamples) within each age decade. Estimates in the ≥ 80 group should be interpreted cautiously due to the small sample size and limited number of events; some measures (e.g., LR−) may be unstable or not estimable when no false‐negative results are observed.

These results suggest that while age adjustment preserved high sensitivity across older age groups, its ability to reduce false positives improved only modestly with advancing age. However, subgroup estimates—particularly in patients aged ≥ 80 years—should be interpreted cautiously because of the small sample size, absence of false‐negative cases in this subgroup, and resulting instability of some diagnostic accuracy estimates. This finding highlights the importance of integrating age‐adjusted thresholds with clinical probability estimates rather than relying on D‐dimer results alone, especially in elderly patients, where D‐dimer elevation is common and nonspecific.

## 4. Discussion

PE remains a significant cause of morbidity and mortality, and its rapid and accurate diagnosis continues to be a challenge in emergency medicine [1, 2]. D‐dimer testing is a widely accepted component of diagnostic algorithms for suspected PE, especially in patients with low or intermediate pretest probability [3, 4, 8]. Even with contemporary probability‐adapted pathways (e.g., YEARS), interpreting D‐dimer values near individualized decision thresholds remains a common real‐world challenge—particularly in older adults. Because structured pretest probability assessment was not consistently available in our retrospective data, direct mapping of our findings to specific guideline pathways is limited. However, D‐dimer levels physiologically increase with age, reducing specificity and often leading to unnecessary imaging in older adults [9]. To mitigate this, AAD thresholds—commonly calculated as age × 0.01 µg/mL for patients ≥ 50 years—have been proposed and validated in large retrospective and prospective studies [6, 9].

In this retrospective analysis of 979 patients aged 50 years and older, we found that AAD preserved high sensitivity (97.5%) while improving specificity (11.8%) compared to the conventional 0.5 µg/mL threshold. These results are consistent with the findings of Douma et al., who demonstrated that the use of AAD significantly increased the number of patients in whom PE could be safely excluded, particularly in older adults [6]. Our results also align with Schouten et al., who, in a meta‐analysis of over 12,000 patients, reported that age‐adjusted thresholds improved specificity while maintaining sensitivity above 97% [7].

A unique contribution of this study is the evaluation of diagnostic performance in a “borderline zone,” defined as D‐dimer values within ±0.1 µg/mL of the AAD threshold. Among the 130 patients in this subgroup, the PE rate was 7.7%, significantly lower than in the nonborderline group (17.9%). However, age‐stratified analysis revealed that the risk of PE increased with advancing age within the borderline group—reaching 28.6% in patients aged ≥ 80. These findings emphasize the importance of interpreting borderline D‐dimer results in the context of patient age and clinical presentation [10]. Accordingly, near‐threshold AAD results should be interpreted cautiously—particularly in older adults—and integrated with overall clinical assessment rather than used in isolation.

We also examined the clinical course of patients who had negative AAD results but were ultimately diagnosed with PE. Among 100 patients who tested negative by AAD but positive by standard D‐dimer, three (3.0%) were found to have PE, including subsegmental or segmental emboli. No in‐hospital complications were documented in the available records; however, postdischarge outcomes and longer‐term safety could not be reliably assessed. These findings are in line with the prospective ADJUST‐PE study by Righini et al., in which only one PE (0.3%) occurred among 331 patients with D‐dimer values below the age‐adjusted threshold, yielding a high NPV of 99.7% [9]. Similarly, Fuchs et al. reported a PE rate of 0.48% in patients between 500 ng/mL and the age‐adjusted cutoff, with an NPV of 99.5% [11]. In our study, this rate was ∼3.0%, consistent with the low event rates reported elsewhere.

Additionally, ROC analysis showed similar diagnostic performance between standard and age‐adjusted thresholds, with AUCs of 0.734 and 0.727, respectively. These values indicate moderate discriminative ability, consistent with D‐dimer’s primary role as a rule‐out test rather than a stand‐alone discriminative tool. This is consistent with findings from Sharp et al., who reported comparable accuracy using AAD in a large, multicenter ED cohort [12]. While the improvement in specificity with AAD may appear modest, its impact on reducing unnecessary imaging is clinically meaningful, particularly in older populations frequently subjected to overtesting.

In most contemporary cohorts, the prevalence of PE among tested patients is below 10% [2, 12]. In our study, the yield was higher (16.5%), likely because only patients who underwent definitive imaging were included. This imaging‐selected design may have introduced verification and spectrum bias by excluding lower‐risk patients with negative D‐dimer results who were not referred for imaging. As a result, the cohort likely had higher clinical suspicion and a higher baseline D‐dimer distribution than broader ED populations evaluated for suspected PE. This selection effect may make sensitivity appear higher and specificity lower and may also affect predictive values. Accordingly, the relatively low specificity observed for AAD in our study should be interpreted primarily as a consequence of the imaging‐selected spectrum and verification process, rather than as reflecting the intrinsic performance of the age‐adjusted threshold in unselected ED populations. Additionally, the proportion of patients undergoing V/Q scintigraphy (14.1%) was higher than in many contemporary cohorts. This likely reflects local practice patterns, the use of V/Q as an alternative in patients with contrast contraindications, and its role as a follow‐up test after nondiagnostic or technically limited CTPA.

Finally, despite the retrospective design and the absence of standardized pretest probability scores, our study provides imaging‐confirmed real‐world ED data and focuses on a practical area of uncertainty: near‐threshold (borderline) AAD results. By analyzing the borderline zone and characterizing AAD‐negative PE cases, we aim to complement probability‐guided diagnostic pathways (e.g., YEARS) by highlighting situations in which additional clinical reassessment may be warranted.

### 4.1. Limitations

This study has several limitations inherent to its retrospective design. First, formal clinical probability scores such as the Wells, Geneva, or YEARS criteria were not routinely documented and therefore could not be incorporated into the analysis. As a result, stratification by pretest probability was not possible. Because D‐dimer and AAD are intended for use within diagnostic pathways restricted to patients with low or intermediate clinical probability, our findings cannot be directly applied to guideline‐recommended probability‐based strategies. Therefore, the results should not be interpreted as supporting AAD use as a stand‐alone rule‐out test.

Second, only patients who underwent definitive imaging (CTPA or V/Q scintigraphy) were included, which may introduce verification and spectrum bias. Patients with low clinical probability and negative D‐dimer results who were managed without imaging were not captured. This imaging‐selected design likely enriched the cohort for patients with higher clinical suspicion, higher baseline D‐dimer values, and higher PE prevalence than would be expected in an unselected ED population. Consequently, sensitivity may appear higher and specificity lower than in broader ED populations, and predictive values may also be affected. Therefore, the diagnostic accuracy estimates should be interpreted as applying primarily to an imaging‐selected cohort rather than to all ED patients evaluated for suspected PE.

Third, the “borderline zone” was defined as an absolute ±0.1 µg/mL window around each patient’s AAD cutoff. This threshold was selected a priori as a pragmatic range to represent values close to the individualized decision boundary and to approximate analytical or reporting variation near the cutoff. Nevertheless, this definition remains partly arbitrary. We did not perform sensitivity analyses using alternative thresholds because the borderline‐zone analysis was exploratory and included only a limited number of PE events (10 PE cases among 130 borderline‐zone patients); narrower or wider windows would likely have produced small subgroups with unstable estimates. Alternative definitions, including relative windows such as ±10% of the individualized cutoff, should be evaluated in future prospective studies. In addition, subgroup findings, especially those for patients aged ≥ 80 years, should be interpreted with caution because of the small number of patients and PE events, as well as the instability of estimates when no false‐negative cases are observed.

Fourth, long‐term clinical follow‐up was not systematically available for the entire cohort. For the three patients with PE despite negative AAD results, chart review showed no documented in‐hospital complications or adverse events at discharge; however, postdischarge outcomes, delayed PE‐related events, recurrent venous thromboembolism, and longer‐term mortality could not be reliably assessed. Therefore, this study cannot establish the longer‐term clinical safety of ruling out PE using AAD beyond the short‐term outcomes documented in the available medical records.

Fifth, we could not reliably determine whether patients had received therapeutic anticoagulation before D‐dimer sampling (e.g., recent DOAC/warfarin use or prehospital heparin). Therapeutic anticoagulation may lower D‐dimer concentrations and increase the risk of false‐negative results; therefore, the direction and magnitude of any resulting bias in our accuracy estimates are uncertain. This limitation may also have contributed to the small number of false‐negative AAD results observed in our cohort. Prospective studies should exclude or analyze recently anticoagulated patients separately.

Finally, data abstraction was performed retrospectively from electronic health records. Although a standardized data collection protocol was used, abstractors were not blinded to the study hypothesis, and no duplicate independent data extraction was performed. Therefore, information bias, incomplete documentation, and inter‐abstractor variability cannot be completely excluded. In addition, PE classification relied on original clinical radiology reports rather than central imaging review. This may have introduced misclassification bias, particularly for small, subsegmental, indeterminate, or borderline findings.

Despite these limitations, our study offers real‐world insights into near‐threshold AAD interpretation and supports the need to integrate AAD results with structured clinical probability assessment rather than relying on D‐dimer results alone.

## 5. Conclusions

In this imaging‐selected retrospective cohort of patients aged 50 years and older with suspected PE, AAD improved specificity compared with the conventional fixed D‐dimer threshold while preserving high sensitivity. However, because the cohort included only patients who underwent definitive imaging and lacked structured pretest probability assessment, these findings should not be interpreted as supporting AAD as a stand‐alone rule‐out strategy. AAD should be applied only within clinical probability‐guided diagnostic pathways. Near‐threshold AAD results, particularly in older adults, are not risk‐free and may warrant further clinical reassessment, repeat testing, or imaging depending on the overall clinical probability. Longer‐term safety after ruling out PE with AAD could not be established in this retrospective study.

## Author Contributions

Cem Yıldırım, Ahmet Fırat Bektaş, and Ertuğ Günsoy conceived the study and designed the study protocol. Cem Yıldırım, Ahmet Fırat Bektaş, and Ahmet Aykut supervised data collection. Cem Yıldırım, Ertuğ Günsoy, and Ahmet Aykut performed data extraction and managed the data, including quality control. Cem Yıldırım, Ahmet Fırat Bektaş, Ahmet Aykut, Mehmet Veysel Öncül, and Ertuğ Günsoy provided statistical advice on study design and analyzed the data. Cem Yıldırım, Ahmet Fırat Bektaş, Ertuğ Günsoy, Ahmet Aykut, Vedat Kırpat, and Mehmet Veysel Öncül drafted the manuscript, and all authors contributed substantially to its revision. Cem Yıldırım takes responsibility for the manuscript as a whole.

## Funding

No funding was received for this manuscript.

## Conflicts of Interest

The authors declare no conflicts of interest.

## Data Availability

De‐identified data and the data dictionary are available from the corresponding author upon reasonable request, subject to institutional approval and applicable ethical and privacy regulations.
